# Blockbuster Laryngeal Mask Airway Versus Endotracheal Tube for Airway Management in Off-Pump Cardiothoracic Surgery: A Randomized Controlled Study

**DOI:** 10.7759/cureus.103946

**Published:** 2026-02-20

**Authors:** Kavya Rajagopal, Bharat Choudhary, Kriti Bhandari, Abhinav Singh, Shikha Soni, Rakesh Karnawat

**Affiliations:** 1 Department of Anaesthesiology, Dr. Sampurnanand Medical College, Jodhpur, IND; 2 Department of Cardiothoracic Surgery, Dr. Sampurnanand Medical College, Jodhpur, IND

**Keywords:** blockbuster lma, endotracheal tube, general anesthesia, hemodynamic response, off-pump cardiothoracic surgery

## Abstract

Background and aims

The increasing use of supraglottic airway devices has led to a shift in airway management practices. BlockBuster® Laryngeal Mask Airway (BLMA; Henan Tuoren Medical Device Co., Ltd., China) is a newer second-generation device, and its use in prolonged surgeries remains limited. Off-pump cardiothoracic surgeries are conventionally performed using endotracheal intubation, which may be associated with significant haemodynamic responses.

Methods

This prospective randomized study included 60 adult patients undergoing elective off-pump cardiothoracic surgeries, allocated into two groups of 30 each. Group A had the airway secured using a Blockbuster LMA, and Group B used an endotracheal tube. Time taken to achieve an effective airway, number of attempts, ease of insertion, intraoperative haemodynamic and ventilatory parameters, and post-operative complications were assessed.

Results

The time taken to achieve effective airway was significantly shorter in Group A compared to Group B (p<0.001). Haemodynamic parameters including heart rate, systolic blood pressure, diastolic blood pressure, and mean arterial pressure were significantly higher in Group B during the immediate period following airway securement (p<0.05). The incidence of post-operative sore throat was significantly higher in the endotracheal tube group (p<0.05). Ventilatory parameters were comparable between the two groups.

Conclusion

Blockbuster LMA is a suitable alternative to the endotracheal tube for airway management in patients undergoing off-pump cardiothoracic surgery, providing quicker airway establishment, better haemodynamic stability, effective ventilation, and fewer post-operative complications.

## Introduction

Airway management is the cornerstone of anaesthesia practice in patients undergoing surgeries under general anaesthesia (GA) [1]. For upper airway management, endotracheal intubation with conventional endotracheal (ET) tube is frequently carried out; however, anaesthetists face significant difficulties when utilising this tube [2].

The first laryngeal mask airway (LMA), the LMA Classic, was designed by Dr. Archie I. J. Brain [3]. Several modifications of LMAs were developed subsequently. A newer second-generation BlockBuster® Laryngeal Mask Airway (BLMA; Henan Tuoren Medical Device Co., Ltd., China), created by Prof. Ming Tian, has unique features that facilitate ventilation and intubation [4]. The BLMA is made up of soft, pliable silicone to avoid trauma. It has a guidance device that directs the tracheal tube towards the laryngeal opening at an angle of 30°, which enhances the rate of successful blind intubation [5]. It can be used as a rescue device for unanticipated difficult intubation and also allows blind and fibre-optic-guided intubation using the specialised BlockBuster ET tube [6]. Thus, BLMA can function both as an airway device and as an aid for intubation. BLMA is a better conduit for oro-tracheal intubation than Fastrach® LMA (Teleflex Incorporated, Amersham, UK) in terms of higher first attempt success rate, lesser time taken for intubation, and lesser post-operative sore throat with additional features like channel for gastric drainage and lower cost. BLMA offers an easy gastric insertion, a smooth gastric inlet and outlet, and a >95° angulation that closely replicates the human anatomical airway [7].

Compared with the ET tube, LMA offers advantages such as easier placement, better haemodynamic stability during induction and emergence, improved airway tolerance, and a lower incidence of post-operative sore throat [3]. For off-pump coronary artery bypass surgery, the objectives of anaesthetic treatment include preserving haemodynamic stability, preserving organ function, enabling early recovery, and providing adequate post-operative analgesia [8].

Although the benefits of LMA, including BLMA, are well established in short-duration and laparoscopic surgeries, data supporting their use in prolonged cardiac surgeries remain limited. The primary objective of this prospective randomized study was to compare the time taken to achieve an effective airway using the BLMA versus an ET tube in patients undergoing off-pump cardiothoracic surgery. Secondary objectives included comparison of peri-airway and intraoperative hemodynamic parameters, ventilatory parameters, and postoperative airway-related complications.

## Materials and methods

Study design, ethics, and patient selection

This study was registered with the Clinical Trials Registry-India (CTRI/2023/08/056266) and was conducted according to Good Clinical Practice standards from August 2023 to January 2024. Approval from the Institutional Ethics Committee of Dr. Sampurnanand Medical College, Jodhpur, Rajasthan, India (NO. SNMC/AcadCТ/ЛЕС/2023/Plan/717) was obtained, and written informed consent was taken from the patients. Sixty patients with American Society of Anaesthesiologists Physical Status (ASA PS) II and III, ages 35 to 65, participated in the current prospective, randomised, open trial and underwent elective fast-track off-pump cardiothoracic surgeries under general anaesthesia [2]. Exclusion criteria exercised were obesity (BMI >30 kg/m²), history of gastroesophageal reflux disease (GERD), high inotropic support pre-operatively, anticipated difficult intubation using Modified Mallampati Grading grade (MPG) III and IV, individuals with severe respiratory conditions, poor left ventricular function (ejection fraction <35%), and potentially fatal arrhythmias or unstable angina pectoris.

Randomisation and preoperative assessment

The randomisation was done on a computer-generated random number chart using block randomization [1:1]. The allocation concealment was done by an opaque, sealed envelope method, opened during the pre-anaesthetic check-up. All patients were randomly allocated into two equal groups: Group A (BLMA group, n=30) or Group B (ET tube group, n=30). Each patient was subjected to detailed history, thorough physical examination, and systemic examination prior to the surgery. Basic demographic data like age, height, and weight were recorded. Routine investigations and any specific test if required were asked for as per institutional guidelines from all patients. Patients were explained in detail about the procedure. Pre-operative fasting as per the Indian Society of Anaesthesiologists (ISA) guidelines was ensured. Patients were advised to continue their regular medications till the morning of surgery, with the exception of angiotensin converting enzyme inhibitors, whose morning dosage was omitted, and antiplatelets, which were ceased five to seven days before the day of surgery.

Intraoperative monitoring

The patients were shifted to the operating room (OR) and were connected to standard multi-channel monitors including heart rate (HR), Electrocardiography (ECG), peripheral oxygen saturation (SpO₂) and non-invasive blood pressure (NIBP), and baseline readings were noted. An 18G IV cannula was used to secure the intravenous line and intravenous fluid balanced salt solution was started slowly. Invasive blood pressure monitoring was started after inserting a femoral arterial cannula under local anaesthesia using ultrasonography guidance while maintaining all aseptic precautions. Continuous cardiac output monitoring was performed by pulse wave analysis using Edward’s Flo Trac system (Edwards Lifesciences, USA).

Anaesthetic technique

Pre-medication was administered using Intravenous lignocaine 2% (1.5 mg/kg), intravenous midazolam (1 mg) and fentanyl intravenously (2 μg/kg). Intravenous propofol (2 mg/kg) was used to induce anaesthesia in a titrated manner after three minutes of pre-oxygenation with 100% oxygen. After confirming adequate bag and mask ventilation, intravenous rocuronium (1.2 mg/kg) was given as the skeletal muscle relaxant and mask ventilation was continued for another 1.5 minute. This was followed by securing the airway by BLMA or ET tube of appropriate size in the respective groups. A third-year anaesthesia resident who had performed >30 insertions of BLMA and endotracheal intubation performed all the airway device insertions in the presence of a senior anaesthesiologist.

Airway management

Group A

Individuals with weight ranges from 30 to 50 kg, 50 to 70 kg, and 70 to 100 kg, respectively, were assigned BLMA sizes 3, 4, and 5 based on their weight. The BLMA was lubricated using the lubricant jelly that was available along with the BLMA and was inserted in the neck extended position.

To successfully insert the BLMA, a maximum of three attempts were allowed, after which the device was considered as a failure and intubation was done using an ET tube, and those patients were excluded from the study.

Group B

In the ET tube group, 7.5 mm cuffed tubes were used for female patients and 8.0 mm cuffed tubes were used for male patients, and intubation was done using conventional laryngoscopy with a Macintosh blade.

Airway assessment parameters

In all groups, the amount of time needed to establish a functional airway, the number of attempts made, and the ease of insertion were recorded. The time interval between picking up and inserting the airway device and the appearance of third square waveform on the capnograph was used to calculate the effective airway time for the BLMA group. For the ET tube, this was calculated from the time of start of laryngoscopy, followed by intubation and the appearance of third square waveform on the capnograph. Ease of device insertion was graded using a five-point Likert scale [9].

Intraoperative management

After confirming the successful positioning of BLMA or ET tube in the respective groups, the devices were fixed and connected to a mechanical ventilator (Dräger Fabius workstation; Drägerwerk AG & Co. KGaA, Germany). To keep the end-tidal CO_2_ within the range of 35-40 mmHg, patients were ventilated using a volume-controlled mode with a tidal volume set at 8 mL/kg and adjustments made to the respiratory rate. Under all aseptic precautions, ultrasound-guided internal jugular vein cannulation was done using a 7 Fr (adult size) triple lumen central venous catheter. Vasopressor infusions were connected to the central line ports and central venous pressure monitoring was started.

Anaesthesia was maintained using inhalational isoflurane (according to minimum alveolar concentration of 1-1.2%) and oxygen. Intravenous rocuronium (0.1 mg/kg) was given intermittently every 30 minutes or on detection of spontaneous breathing on the capnogram. Intravenous fentanyl (2 µg/kg) was given prior to sternotomy and followed by a dose of 1 µg/kg repeated every hour for analgesia. Intravenous paracetamol (15 mg/kg) was also given at the end of surgery to provide post-operative analgesia. Parameters including HR, blood pressure (BP; systolic, diastolic and mean arterial pressure (SBP, DBP, MAP, respectively)), SpO₂, and peak airway pressure were measured. HR and BP were monitored at zero, one, three, five, and 10 minutes after airway insertion and thereafter every 30 minutes until closure and shifting to intensive care unit.

Postoperative management

After surgery, patients were shifted to the cardiothoracic ICU with the respective airways (BLMA or ET tube) in situ and with full monitoring. The patients were monitored further in the ICU. Extubation was planned if the patients were cooperative and alert, had oxygen saturation (SpO₂) ≥98%, FiO₂<50% and end-tidal CO₂<45 mmHg, normal arterial blood gas analysis, stable haemodynamics with few inotropes, and no arrhythmias or hypothermia. After proper oral and oropharyngeal suctioning, patients were extubated. Reversal agent intravenous sugammadex (2 mg/kg) was given before extubation. Following extubation, patients were monitored and complications following surgery (cough, hoarseness of voice, and sore throat) were documented if present, by an independent observer. Patients requiring prolonged mechanical ventilation during or after surgery were excluded from the study.

Sample size calculation and statistical analysis

A previous study by Ng et al. [10] was used for sample size calculation, which showed a minimum requirement of 21 subjects per group. With a 90% research power and an alpha error of 0.05, this was increased and rounded to 30 patients per group to increase the strength of study.

All statistical analyses were performed using Jamovi Solid (Jamovi (Version 2.6.44) (Computer Software). Retrieved from https://www.jamovi.org). Analyses were conducted using a per-protocol approach, as all randomised participants received the allocated intervention and were included in the final analysis. Normality of continuous variables was assessed using Q-Q plots and the Shapiro-Wilk test, while homogeneity of variances was checked with the Levene’s test. Continuous variables conforming to normal distribution were expressed as mean ± standard deviation (SD). Between-group comparisons for continuous data such as age, weight, height, time to achieve airway, and hemodynamic parameters were made using the independent samples t-test, and results were reported as mean differences with 95% confidence intervals (CI).

For intraoperative hemodynamic variables (HR, SBP, DBP, MAP) measured across multiple time points, repeated-measures analysis of variance (ANOVA) was applied to test within-subject time effects and time × group interactions. The Greenhouse-Geisser correction was used if the assumption of sphericity was violated. Partial eta squared (η²) was reported to indicate effect sizes.

Categorical variables including gender distribution, number of attempts, ease of insertion, and postoperative complications were presented as frequencies and percentages. Chi-square test was used for group comparisons; however, if the Chi-square assumption was violated (>20% of cells had expected counts less than five), then Fisher’s exact test was applied for 2×2 contingency tables, and the Chi-square test with continuity correction was used for larger tables. A two-tailed p-value <0.05 was considered statistically significant.

## Results

**Figure 1:**
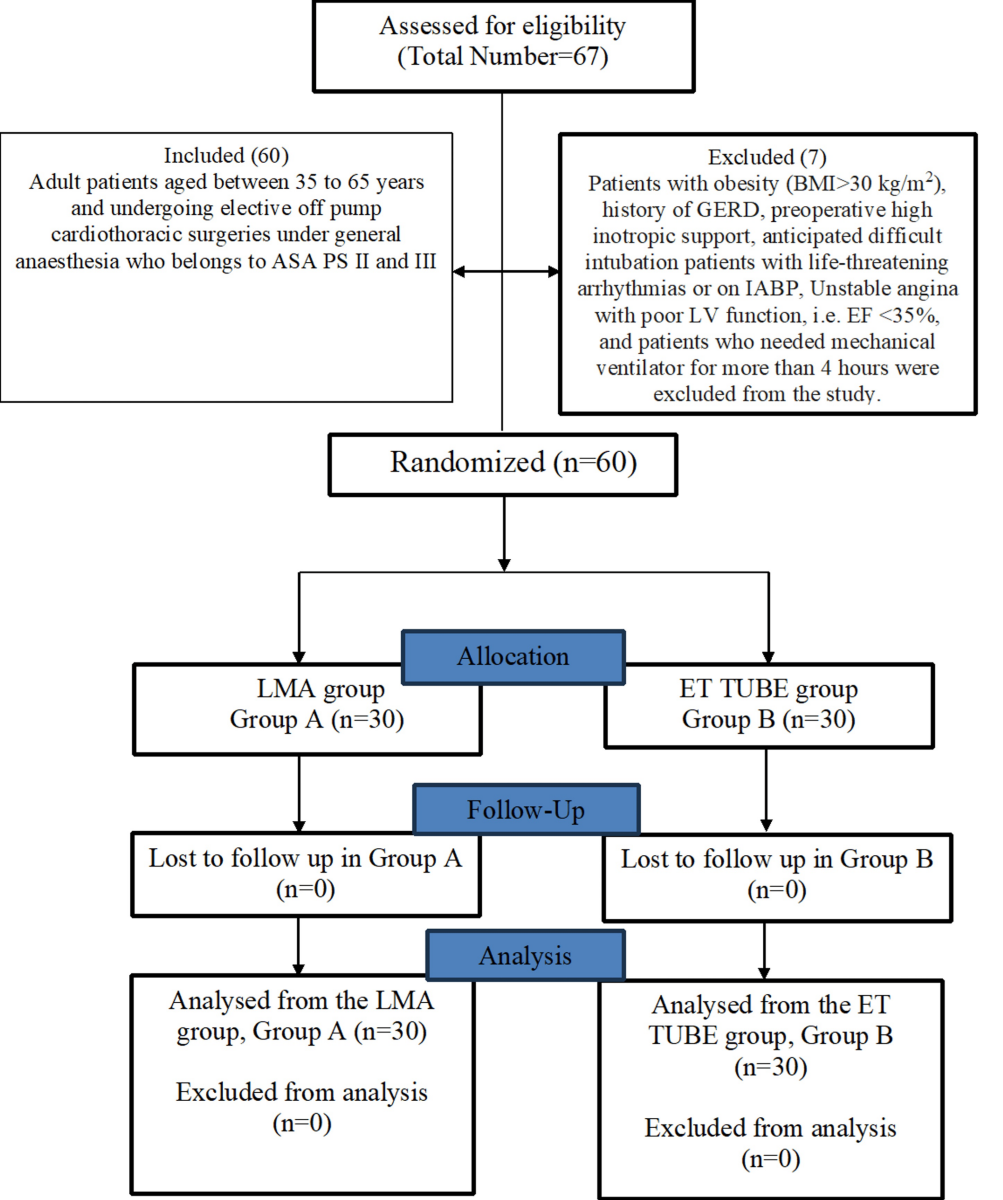
CONSORT study flow chart Of 67 patients assessed, 60 were included and randomized to the LMA group (n=30) or ET tube group (n=30). Seven patients were excluded based on predefined criteria. There were no losses to follow-up or exclusions from analysis, and all randomized patients were analyzed. CONSORT: Consolidated Standards of Reporting Trials; LMA: Laryngeal mask airway; ET tube: Endotracheal tube; ASA PS: American Society of Anaesthesiologists Physical Status; GERD: Gastro oesophageal reflux disease; BMI: Body mass index; LV: left ventricle; EF: ejection fraction; n: number of patients.

The eligibility of 67 patients was evaluated. According to the exclusion criteria, seven patients were eliminated. Sixty patients were enrolled and randomised into two groups, with 30 patients each assigned to the Blockbuster LMA group (Group A) and the endotracheal tube group (Group B). Every patient who was randomised got the assigned intervention and was part of the final analysis as per protocol. Following randomisation, no patients were lost to follow-up or eliminated. The Consolidated Standards of Reporting Trials (CONSORT) [11] flow diagram depicting patient enrolment, allocation, follow-up, and analysis is shown in Figure 1.

Baseline demographic and haemodynamic characteristics

Both the groups were comparable in terms of demographic profile (Table 1).

**Table 1:** Demographic data Data are expressed as mean ± SD or number. Independent t-test and Chi-square test were used. A p-value <0.05 was considered statistically significant and denoted by an asterisk (*).

Parameter	Group A	Group B	Mean Difference (95% CI)	Statistic (df)	p-value
Age (years)	53.6 ± 12	54.1 ± 11	0.43 (−5.53, 6.40)	t = 0.145 (58)	0.885
Gender (M/F)	18/12	20/10	–	χ² = 0.287 (1)	0.592
Weight (kg)	62.6 ± 7.3	64.0 ± 10.3	1.3 (−3.3, 5.9)	t = 0.565 (58)	0.573
Height (cm)	160.7 ± 6.9	161.4 ± 7.1	0.7 (−2.9, 4.3)	t = 0.386 (58)	0.7

**Figure 2:**
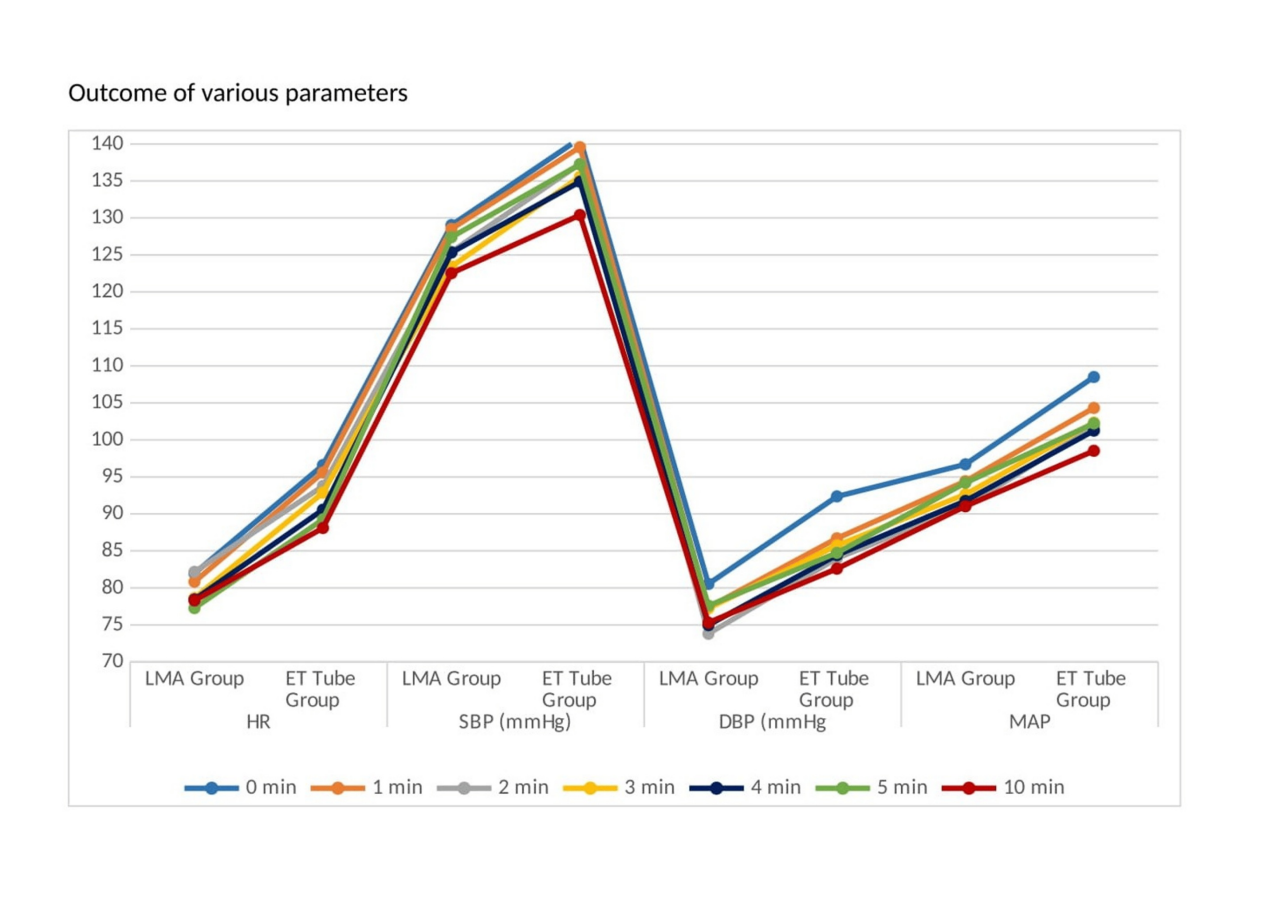
Outcome of the various hemodynamic parameters Comparison of the peri-intubation hemodynamic parameters between the Blockbuster laryngeal mask airway (LMA) group and endotracheal tube (ET) group. Changes in heart rate (HR; beats/min), systolic blood pressure (SBP; mmHg), diastolic blood pressure (DBP; mmHg), and mean arterial pressure (MAP; mmHg) were recorded at baseline (0 min) and at 1, 2, 3, 4, 5, and 10 minutes after securing the airway. The ET tube group demonstrated higher haemodynamic responses compared to the LMA group during the immediate post-airway period.

The baseline haemodynamic parameters, such as SBP, DBP, MAP, and HR, were similar in both groups (Figure 2).

In contrast, intraoperative haemodynamic values measured at predetermined time intervals exhibited statistically significant differences immediately following airway stabilisation, with elevated values noted in Group B. Repeated-measures ANOVA with Greenhouse-Geisser correction revealed significant within-subject time effects and time × group interactions, with partial eta squared values indicating moderate effect sizes for HR and large effect sizes for mean arterial pressure, suggesting that the observed differences were not only statistically significant but also clinically relevant (Table 2).

**Table 2:** Intra-operative heart rate and mean arterial pressure For heart rate, repeated-measures ANOVA using the Greenhouse–Geisser correction due to violation of sphericity demonstrated a significant within-subjects time effect (F(3.31, 191.78) = 7.19, p<0.001, η² =0.110) and a significant time × group interaction (F(3.31, 191.78) =5.55, p<0.001, η² =0.087). For mean arterial pressure, Greenhouse–Geisser–corrected repeated-measures ANOVA showed a significant within-subjects time effect (F(5.20, 301.5)=9.03, p<0.001, η² =0.135), along with a significant time × group interaction (F(5.20, 301.5) = 6.22, p<0.001, η² =0.097).

Time point	Heart rate mean difference (95% CI)	t (df)	P-value	Mean arterial pressure mean difference (95% CI)	t (df)	p-value
Before induction	−2.0 (−8.6, 4.6)	−0.59 (58)	1	0.8 (−5.0, 6.6)	0.28 (58)	1
0 min	−14.6 (−21.3, −8.0)	−4.67 (58)	0.002*	−11.8 (−18.9, −4.7)	−3.31 (58)	0.193
1 min	−14.8 (−19.7, −9.9)	−6.02 (58)	<0.001*	−9.9 (−16.3, −3.5)	−3.04 (58)	0.42
2 min	−11.6 (−19.6, −3.6)	−3.78 (58)	0.045*	−10.8 (−17.0, −4.7)	−3.75 (58)	0.049*
3 min	−14.2 (−18.9, −9.6)	−6.33 (58)	<0.001*	−9.7 (−15.9, −3.6)	−3.26 (58)	0.223
4 min	−12.1 (−16.5, −7.7)	−5.46 (58)	<0.001*	−9.5 (−15.9, −3.1)	−2.94 (58)	0.567
5 min	−12.0 (−16.8, −7.2)	−5.66 (58)	<0.001*	−8.0 (−14.3, −1.7)	−2.47 (58)	1
10 min	−9.8 (−15.4, −4.2)	−3.55 (58)	0.092	−7.5 (−13.7, −1.3)	−2.41 (58)	1

Airway management parameters

With a mean time of 36.5 seconds against 73.7 seconds in group B (p<0.001) and a mean difference of 37.2 seconds, group A's time to create an effective airway was significantly shorter (Table 3).

**Table 3:** Time taken to achieve effective airway among both groups The time to achieve an effective airway was markedly shorter with the Blockbuster LMA (36.5 ± 11.2 sec) compared to the endotracheal tube (73.7 ± 21.2 sec), with a mean difference of 37.2 seconds (95% CI 28.4–46.0, p<0.001). A p-value <0.05 was considered statistically significant and denoted by an asterisk (*).

Time taken to achieve effective airway (sec.)	Group A	Group B	Mean Difference (95% CI)	p-value
	36.5 ± 11.2	73.7 ± 21.2	37.2 (28.4, 46)	<0.001*

Post-operative complications were also evaluated, with a significantly lower incidence of sore throat in group A (10%) compared to group B (40%) (p=0.007). Other complications like cough and hoarseness of voice were comparable among both groups (Table 4).

**Table 4:** Comparing post-operative complications among both groups Post-operative complications showed clear differences between the two groups. The incidence of sore throat was significantly higher with the endotracheal tube (40%) compared to the Blockbuster laryngeal mask airway (LMA; 10%), χ²(1) =7.2, p=0.007, with a moderate effect size (0.346, 95% CI 0.10–0.55). In contrast, cough and hoarseness of voice were infrequent in both groups, with no statistically significant differences as assessed by Fisher’s Exact Test. Categorical variables are expressed as number (%). The Chi-square test was used for comparisons where expected cell counts were adequate; Fisher’s Exact test was applied when expected cell frequencies were <5. A p-value <0.05 was considered statistically significant and denoted by an asterisk (*).

Parameter	Group A	Group B	Statistic (df)	P-value	Effect Size (95% CI)
Sore throat – Present	3 (10%)	12 (40%)	χ² = 7.2 (1)	0.007*	0.346 (0.10–0.55)
Absent	27 (90%)	18 (60%)	–	–	–
Cough – Present	3 (10%)	5 (16.7%)	Fisher’s Exact	0.706	–
Absent	27 (90%)	25 (83.3%)	–	–	–
Hoarseness of voice – Present	1 (3.3%)	4 (13.3%)	Fisher’s Exact	0.353	–
Absent	29 (96.7%)	26 (86.7%)	–	–	–

Ventilatory parameters

The ventilatory parameters including tidal volume, minute ventilation and end tidal CO₂ were studied at different time intervals and no clinically or statistically significant differences were noted between the groups.

## Discussion

BLMA characteristics and haemodynamic considerations

The BLMA is a second-generation supraglottic airway device with an anatomically preformed airway tube designed to optimise pharyngeal alignment, provide high oropharyngeal sealing pressure, and facilitate ventilation as well as blind or fiberoptic-guided tracheal intubation [12-14]. Introduced in 2012 in China by Professor Ming Tian, the device incorporates an integrated gastric drainage channel that may help reduce the risk of gastric insufflation and aspiration by allowing gastric decompression [4,13].

In the present study, standard preoperative fasting was ensured in all patients, and gastric suctioning was routinely performed through the drainage channel of the BLMA after device placement. The patients undergoing off-pump cardiac surgeries are at risk of the occurrence of a further cardiac event in the perioperative period. Hence, maintenance of haemodynamic stability during the induction of anaesthesia and while securing the airway is mandatory. Therefore, any increase or decrease in BP or HR, which may occur during laryngoscopy, should be avoided in them. In such a scenario, newer devices like BLMA can play a good role, which have shown better haemodynamics and safety compared to the ET tube [15,16]. This study provides a detailed analysis of various parameters, including demographic data, intraoperative and post-operative variables, and associated complications. 

Comparison with existing literature

Comparison of insertion characteristics revealed that both devices were generally successful on the first attempt, with no significant difference between groups (86.7% for BLMA vs. 93.3% for ET tube, p=0.671). However, the ease of insertion was better in group A, with a higher percentage of patients belonging to Likert scale-1 (very easy) (73.33%) compared to group B (46.66%), though this did not reach statistical significance (Chi-square = 7.25, p=0.06). In Group A (36.5 ± 11.2 sec), it took far less time to establish a functional airway compared to Group B (73.667 ± 21.2 sec), with a highly significant p-value of <0.001. A prospective randomised trial done on the Baska® Mask (P.J. Healthcare Pvt. Ltd., India) versus ET tube in laparoscopic cholecystectomy surgery showed the same result, wherein the Baska group's effective airway was achieved in shorter time (26.6 ± 4.7 sec) compared to the ET tube group (47.2 ± 11.8 sec; p<0.001) [13]. This finding emphasises the efficiency of LMA in quickly establishing a secure airway, which is crucial in emergency and time-sensitive situations [13,14]. This can be attributed to the absence of direct laryngoscopy, minimal airway manipulation, and the supraglottic positioning of the device which allow rapid and easy insertion compared to conventional laryngoscopy and endotracheal intubation.

After securing the airway, the study of HR, SBP, DBP, and MAP at zero, one, two, three, four, five, and 10 minutes revealed greater values in group B than in group A. This difference was statistically significant (p<0.05).These results are consistent with findings from a study on better haemodynamic profile of LMA insertion compared to laryngoscopy and tracheal intubation [16]. This study proved that use of LMA is associated with reduced haemodynamic responses compared to ET tube (p<0.001). The study conducted by Shah et al. [3], using ProSeal laryngeal mask airway as a substitute for the conventional ET tube in order to secure the upper airway in patients receiving coronary artery bypass grafting, demonstrated a similar result. The haemodynamic parameters such as MAP and HR showed lower values in the ProSeal LMA group than the ET tube group (p<0.05). In a prospective randomized study involving patients undergoing coronary artery bypass grafting, airway management with a LMA was associated with significantly less increase in MAP and HR during induction compared to tracheal intubation performed using either direct laryngoscopy or an intubating LMA, suggesting improved haemodynamic stability with supraglottic airway use in patients with coronary artery disease compared to the other groups (p<0.05) [15].

These findings are indicative of more cardiovascular stress associated with ET tube. As a supraglottic airway device, the laryngeal mask airway avoids direct laryngoscopy and tracheal instrumentation, which is known to reduce airway stimulation during insertion [15-17].

This study found no significant differences between the two groups concerning oxygen saturation (SpO_2_), peak airway pressure, minute ventilation, end tidal CO_2_, and tidal volume. Similar findings were found in the study by Shah et al. [3] on ProSeal LMA as a substitute for a regular ET tube in securing upper airway in patients undergoing beating-heart coronary artery bypass grafting. There was no statistically significant difference in the respiratory parameters between the ProSeal LMA and ET tube groups at any of the evaluation time points (P>0.05), according to the evaluation of respiratory measures such SpO_2_, pCO_2_, positive airway pressure (PAP), and lung compliance. In a different study, ProSeal LMA and ET tube were compared in patients undergoing laparoscopic procedures under general anaesthesia [18]. This study also revealed that both groups (ProSeal LMA group and ET tube group) maintained perioperative SpO_2_ along with a comparable value of end tidal CO_2_ (maximal concentration of carbon dioxide (CO_2_) at the end of an exhaled breath), while the PAP was also statistically insignificant in both groups. In this selected patient population undergoing fast-track off-pump cardiothoracic surgery, the BLMA demonstrated comparable ventilatory performance to the ET tube.

With a p-value of 0.007, the incidence of post-operative sore throat was substantially lower in group A (10%) than in group B (40%). Even though the number of patients who complained of post-operative cough and hoarseness of voice were more in the group B, it was not statistically significant. A prospective randomized trial on the Baska mask versus the ET tube in laparoscopic surgery [9], showed that the ET tube group was associated with a higher incidence of post-operative complications including cough, trauma, spasm, dysphonia, and sore throat, compared to the Baska mask group, a result similar to our study. The ProSeal group experienced less post-operative problems than the ET tube group, according to a study by Shah et al. [3], when ProSeal LMA was used as an alternative to traditional ET tube in securing the upper airway in patients having beating-heart coronary artery bypass grafting. The lesser incidence of post-operative complications with BLMA could be because of its positioning in the supraglottic region. But it was observed that the patients in whom BLMA was used had complaints of cough and hoarseness of voice in the post-operative period, even though it was statistically not significant. This could be due to the airway oedema caused by the inflated BLMA.

Clinical implications and future directions

In this prospective study, we concluded that the use of BLMA provided safer anaesthesia (better haemodynamic stability and quicker achievement of airway) and lesser post-operative complications, making it a potentially favourable option in terms of procedural efficiency and patient comfort. Therefore, it paves way for an alternative airway management approach compared to conventional ET tube, in patients undergoing off pump cardiothoracic surgery when used by an experienced anaesthesiologist. This study is distinctive as it evaluates BLMA versus ET tube in prolonged cardiac surgeries, in which haemodynamic stability is of utmost importance. Moreover, this evaluation was within an Indian population that remains inadequately explored.

Limitations

The small sample size may limit generalisability. The open-label design, despite rigorous randomisation, may introduce bias, and subjective outcomes such as ease of insertion, time to secure the airway, and post-operative sore throat are susceptible to observer and performance bias. As gastric regurgitation and aspiration were not evaluated, the findings cannot be extrapolated to patients at higher aspiration risk. Airway insertions performed by a single third-year anesthesia resident during routine clinical rotation may further limit generalisability and introduce operator-specific bias, particularly for newer devices such as the BLMA. Additionally, the study was confined to fast-track off-pump coronary artery bypass grafting patients, and the results may not apply to cases where fast tracking is not feasible.

## Conclusions

We concluded that the use of BLMA ensured quicker achievement of effective airway, produced better haemodynamic stability, and lesser post-operative complications (sore throat) as compared to the ET tube. In other words, the BLMA provided safer anaesthesia and fewer post-operative complications, making it a potentially favourable option in terms of procedural efficiency and patient comfort. Therefore, it forges a new path towards an alternative airway management to the conventional ET tube in patients undergoing off-pump cardiothoracic surgery in a selected patient population.

## References

[1] Kumar B, Gupta B, Thakur A, Rana S, Garg M, Nehria M (2022). Comparative evaluation of airway dynamics in patients undergoing laparoscopic cholecystectomy under general anaesthesia with controlled ventilation using ProSeal laryngeal mask airway, I-Gel™ and Baska mask. Indian J Anaesth.

[2] Kalpana Shah (2021). Fast tracking in off pump CABG with supraglottic airway and TIVA. JCCC.

[3] Shah K (2017). ProSeal laryngeal mask airway as an alternative to standard endotracheal tube in securing upper airway in the patients undergoing beating-heart coronary artery bypass grafting. Ann Card Anaesth.

[4] Zhu Xiaojun, Zhu Hairong, Lu Lei (2015). The clinical application of Blockbuster laryngeal mask airway introducing endotracheal tube. Hainan Med J.

[5] Raiger LK, Sharma B, Gehlot RK, Dhania S, Meena HK (2022). A comparison of tracheal intubation with Ambu® AuraGainTM, Fastrach® and BlockBuster® laryngeal mask airway: a randomised clinical trial. J Clin Diag Res.

[6] Khare A, Awana P, Thada B, Mathur V, Kumar P (2022). A randomized comparative study to observe the safety and efficacy of I gel and blockbuster laryngeal mask airway used in patients undergoing short surgical procedure under general anesthesia. Indian Anaesth Forum.

[7] Tripathi MK, Tiwari T, Naithani B, Upadhyaya DN, Singh PR, Tripathi I (2023). Clinical utility of i-gel(®) and BlockBuster™ supraglottic devices for airway management in postburn injury contracture neck patients under general anesthesia: a randomized controlled trial. Int J Crit Illn Inj Sci.

[8] Hemmerling TM, Romano G, Terrasini N, Noiseux N (2013). Anesthesia for off-pump coronary artery bypass surgery. Ann Card Anaesth.

[9] Likert R (1932). A technique for the measurement of attitudes. Arch Psychol.

[10] Ng CC, Sybil Shah MH, Chaw SH, Mansor MB, Tan WK, Koong JK, Wang CY (2021). Baska mask versus endotracheal tube in laparoscopic cholecystectomy surgery: a prospective randomized trial. Expert Rev Med Devices.

[11] Schulz KF, Altman DG, Moher D (2010). CONSORT 2010 statement: updated guidelines for reporting parallel group randomized trials. Ann Intern Med.

[12] Premkumar KG, Vijayalakshmi H, Shanthini S (2023). A comparative study of supraglottic airway devices Classic LMA, ProSeal LMA, and BlockBuster LMA in adult patients undergoing short surgical procedures. Int J Pharm Clin Res.

[13] Endigeri A, Ganeshnavar A, Varaprasad B, Shivanand YH, Ayyangouda B (2019). Comparison of success rate of BlockBuster(®) versus Fastrach(®) LMA as conduit for blind endotracheal intubation: a prospective randomised trial. Indian J Anaesth.

[14] Kaur K, Verma V, Kumar P (2024). Comparison of the LMA BlockBuster and intubating LMA as a conduit to blind tracheal intubation. J Anaesthesiol Clin Pharmacol.

[15] Bennett SR, Grace D, Griffin SC (2004). Cardiovascular changes with the laryngeal mask airway in cardiac anaesthesia. Br J Anaesth.

[16] Jarineshin H, Kashani S, Vatankhah M, Abdulahzade Baghaee A, Sattari S, Fekrat F (2015). Better hemodynamic profile of laryngeal mask airway insertion compared to laryngoscopy and tracheal intubation. Iran Red Crescent Med J.

[17] Simon LV, Torp KD (2024). Laryngeal mask airway. StatPearls [Internet].

[18] Saraswat N, Kumar A, Mishra A, Gupta A, Saurabh G, Srivastava U (2011). The comparison of Proseal laryngeal mask airway and endotracheal tube in patients undergoing laparoscopic surgeries under general anaesthesia. Indian J Anaesth.

